# Clinical Characteristics and Visual Outcomes of Patients Hospitalized for Ocular Trauma in Shandong Province, China

**DOI:** 10.1155/2020/5826263

**Published:** 2020-04-15

**Authors:** Fangnan Duan, Xiunian Chen, Sai Zhang, Xiaolin Qi, Weiyun Shi, Hua Gao

**Affiliations:** ^1^Medical College of Qingdao University, Qingdao 266071, China; ^2^Shandong Eye Hospital, Shandong Eye Institute, Shandong First Medical University, Shandong Academy of Medical Sciences, Jinan 250021, China; ^3^State Key Laboratory Cultivation Base, Shandong Provincial Key Laboratory of Ophthalmology, Shandong Eye Institute, Shandong First Medical University, Shandong Academy of Medical Sciences, Qingdao 266071, China

## Abstract

**Purpose:**

To analyze the clinical characteristics and visual prognoses of patients with ocular trauma treated in Shandong Eye Hospital.

**Methods:**

The inpatient data of patients with eye injuries hospitalized in our institution from January 2014 to December 2018 were retrospectively reviewed, including demographic information, types of trauma, causes of injury, treatment, and initial and final visual acuities.

**Results:**

A total of 1,425 patients (1,622 eyes), aged 39.5 ± 18.5 years, were included. The ratio of male to female was 5.3 : 1. Of the mechanical eye injuries, there were 490 (34.4%) open-globe injuries and 454 (31.9%) closed-globe injuries. Nonmechanical eye injuries had 426 patients (29.9%), while 55 patients (3.9%) had adnexal injuries. Over a half of the traumas were work-related (51.1%, 728 patients). Most patients were treated with surgical intervention (1,404 eyes, 87.9%). There were significant differences in the final visual acuities between open-globe injuries and closed-globe injuries (*P* < 0.001), as well as between mechanical injuries and nonmechanical injuries (*P* < 0.001). The final visual acuity was closely correlated with the initial visual acuity (Spearman's correlation coefficient = 0.618, *P* < 0.001) and the OTS score (Spearman's correlation coefficient = 0.691, *P* < 0.001).

**Conclusion:**

Ocular trauma usually occurs in young and middle-aged men and in the workplace in Shandong Province. The proportion of nonmechanical injuries is high, and the prognosis is poor. A comprehensive understanding of the characteristics of ocular trauma is useful for blindness prevention and treatment.

## 1. Introduction

Ocular trauma is a major preventable cause of permanent visual impairment. There are approximately 55 million eye injuries each year, among whom 750,000 requires hospitalization around the world [1]. The annual incidence of hospitalized ocular trauma is between 8.6 and 27.7 per 100,000 population in China [2, 3]. Ocular injuries, especially those with poor prognosis, have increased economic burdens on patients, their families, and society [4, 5]. To eliminate the occurrence of eye injuries, effective prevention and timely intervention seem to be particularly important.

Shandong Province, with 106,000,000 permanent residents, is one of the most populous areas in China. However, the epidemiological data of eye injuries in the province are lacking. The aim of this study was to provide a basis for the prevention of ocular trauma in Shandong by retrospectively investigating the clinical characteristics of eye injuries and probing the prognostic factors associated with visual outcomes.

## 2. Materials and Methods

Data from all patients with the diagnosis of ocular trauma who were admitted to Shandong Eye Hospital from 1 January 2014 to 31 December 2018 were collected from electronic medical records by trained researchers. Eye injury-related hospitalizations were identified by using the International Classification of Disease, 10th Revision, Clinical Modification. Each patient was included only once.

Demographic and clinical data of all patients were entered into a computerized database separately by two researchers and made available for later review, including patient age, gender, place of residence, occupation, laterality of the injured eye, classification and location of the injury, cause of the injury, time of seeking medical advice, surgical procedure, and initial and final visual acuities. They were used for analysis after checks. If a specific data field was not available for a patient, the patient was excluded from that particular assessment.

According to the Birmingham Eye Trauma Terminology system [6], mechanical injuries were classified as open-globe injuries and closed-globe injuries. For the open-globe injuries, zone I was confined to the cornea and corneoscleral limbus, zone II was 5 mm posterior to the corneoscleral limbus, and zone III was posterior to zone II [7]. We utilized the ocular trauma score to evaluate the severity of ocular trauma [8]. The initial visual acuity was the best corrected Snellen visual acuity (BCVA) at the time of presentation, while the final visual acuity was obtained upon the most recent outpatient visit.

Statistical analyses were performed using Version 25.0 SPSS (IBM Corporation, Armonk, NY, USA). Continuous data that conformed to a normal distribution were reported as mean ± standard deviation. Categorical data were shown as either percentage or rate. Frequency analyses were performed using Pearson's chi-squared test. Correlation analyses (between the initial and final visual acuities and OTS and final visual acuities) were performed using Spearman's test. *P* < 0.05 was considered statistically significant.

## 3. Results

**Figure 1:**
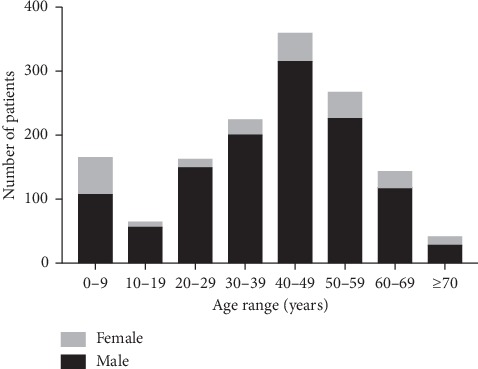
Frequency of ocular trauma by age and gender.

A total of 1,622 eyes (1,425 patients) were included. Among these patients, 1,199 (84.1%) were male and 226 (15.9%) were female in a ratio of 5.3 : 1. The mean patient age was 39.5 ± 18.5 years, ranging from 1 to 92 years. Ocular trauma was mostly encountered at the age of 40–49 years (359 patients, 25.2%), followed by the age of 50–59 years (267 patients, 18.7%) and 30–39 years (224 patients, 15.7%) (Figure 1). Compared with urban areas (276 patients, 19.4%), the injuries occurred more frequently in rural areas (1,149 patients, 80.6%). Farm workers accounted for 34.7% of patients (494 cases), followed by workers (421 patients, 29.5%) and students (164 patients, 11.5%).

As shown in Table 1, open-globe injuries (490 patients with 495 eyes, 34.4%) were the most common type of injuries, followed by closed-globe (454 patients with 476 eyes, 31.9%), thermal/chemical (426 patients with 596 eyes, 29.9%), and adnexal (55 patients with 55 eyes, 3.9%) injuries. Among 490 open-globe injuries, zone I was affected more commonly (327 patients, 66.7%) than zone III (92 patients, 18.8%) and zone II (71 patients, 14.5%). Penetrating injuries mostly occurred in zone I (224 patients, 80.3%), whereas rupture was most frequently observed in zone III (56 patients, 54.9%).

**Figure 2:**
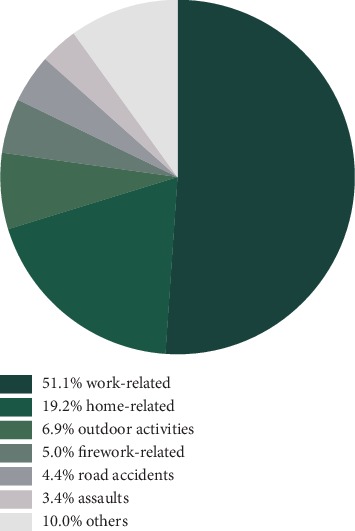
Causes of eye injuries.

The percentage of work-related injuries was 51.1% (728 patients), higher than home-related injuries (273 patients, 19.2%) (Figure 2 ). For the former, the most common causing agents were metal (212 patients, 29.1%), lime/plaster (93 patients, 12.8%), and wood (90 patients, 12.4%). For the latter, the major reasons were cutting by metal (88 patients, 32.2%), falling (46 patients, 16.8%), and splashing of household alkaline cleaning agents (24 patients, 8.8%). Among 426 nonmechanic injuries, the percentage of work-related injuries was 81.7% (348 patients), higher than home-related injuries (56 patients, 13.1%). For the former, the most common causing agents were lime/plaster (93 patients, 26.7%), high temperature metal liquid (86 patients, 24.7%), and sodium hydroxide (42 patients, 12.1%). Only 90 patients (6.3%) had eye protection when the traumatic event happened.

The time interval from injury to presence at the emergency room was also evaluated. Approximately 54.5% of patients did not receive professional medical intervention within 24 hours. There was significant difference in the final visual acuity between patients receiving treatment within and after 24 hours (*P* < 0.01; Pearson's chi-squared test).

Except 22 patients (25 eyes) who withdrew from the management for economic reasons, 181 patients (193 eyes, 12.1%) chose conservative treatment with medications, and 1,222 patients (1,404 eyes, 87.9%) underwent surgical intervention. Debridement and suture (540 eyes, 33.8%) and amniotic membrane transplantation (445 eyes, 27.9%) were major surgical procedures (Table 2). In 18 eyes (1.1%) with NLP, enucleation was performed due to uncontrolled endophthalmitis and severe ocular rupture.

The initial visual acuity was ≥20/40 in 184 eyes (11.3%), between 20/50 and 20/200 in 405 eyes (25.0%), between 19/200 and 1/200 in 347 eyes (21.4%), LP/HM in 445 eyes (27.4%), and NLP in 57 eyes (3.5%). The final visual acuity was ≥20/40 in 396 eyes (24.4%), between 20/50 and 20/200 in 438 eyes (27.0%), between 19/200 and 1/200 in 351 eyes (21.6%), LP/HM in 257 eyes (15.8%), and NLP in 34 eyes (2.1%), closely correlated with the initial visual acuity (Spearman's correlation coefficient = 0.618, *P* < 0.001).

**Figure 3:**
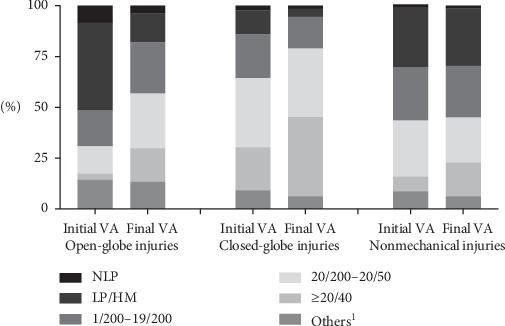
A comparison of the initial visual acuity and the final visual acuity in different types of injuries. ^1^Others: patients cannot receive the visual acuity examination due to the young age or serious symptoms.

As shown in Figure 3, the final visual acuity in patients with closed-globe injury was better than those with open-globe injury (*P* < 0.001, Pearson's chi-squared test). Significantly higher final visual acuity was also found in patients with mechanical injury and those with nonmechanical injury (*P* < 0.001, Pearson's chi-squared test).

The average OTS of mechanical injuries was 70.1 ± 21.4. The final visual acuity was correlated closely with the OTS score (Spearman's correlation coefficient = 0.691, *P* < 0.001) (Table 3).

## 4. Discussion

Ocular trauma has ranked among the most common reasons for hospitalization in the specialty of ophthalmology in industrialized nations [9]. Severe eye injuries may destroy the global structure and induce loss of visual function, which requires effective prevention and therapeutic intervention for reduction of long-term sequelae on the basis of a comprehensive understanding of the characteristics of ocular trauma.

In comparison with previous reports from other areas of China [2, 3, 10, 11], males accounted for a higher proportion in patients suffering ocular trauma than females in the current study. The ratio of male to female was also significantly higher than the data in a report from the United States (5.3 : 1 versus 1.46 : 1) [12]. This difference may be due to the higher proportion of males in China. In terms of age distribution, most of our traumatized patients were 20 to 69 years old, particularly between 40 and 49 years old. The average age of American patients with ocular trauma was 49.4 ± 25.2 years old, with 31.9% of patients being over 65 years old, which was higher than 39.5 ± 18.5 years old in this study. The reasons may be discrepancies in occupation, outdoor exposure, and defection of laws and regulations concerning occupational safety and health [13, 14]. Although China has entered the era of population aging, the process is comparatively slow, and outdoor activities for the elderly may be comparatively limited. Correspondingly, the rate of ocular trauma in the population over 65 years old does not appear to be high. Another phenomenon is higher incidence of female children eye injuries compared with other periods. This may be related to increasing female birth rate in the past decades in our region.

Compared with other studies [12, 15, 16], the proportion of nonmechanical ocular trauma was higher in this study. Shandong Eye Hospital is a major tertiary eye hospital in Shandong Province. Patients with chemical/thermal eye injury in Shandong Province and neighboring provinces are usually referred to our hospital. On the other hand, China's industrialization continues, but many manufacturing processes still rely on manual operations, which maximizes the probability of workers coming into contact with hazardous chemicals. In addition, regular maintenance of equipment and safety of working environment may not be adequately guaranteed in small businesses. Lack of pre-job safety training and protective equipment for dangerous posts makes it more possible to have chemical/thermal injury during work. It was reported that the wearing eye-protection devices can reduce the incidence of ocular trauma with a high probability [17–19], while as few as 6.3% of workers using safety glasses in this study. Many of these injuries could be prevented with the proper use of safety glasses or other protective equipment.

In the current analysis, about half of the eye injuries were related to occupation with a proportion higher than developed countries like the United States and Australia [12, 15]. The predominant reason of eye injuries in these two countries was falling at home in the elderly population. This difference may be associated with quality of work environment and population structure. The percentage of workers in the primary and secondary industries in Shandong Province is relatively high, and meanwhile, the level of mechanical automation is not as good as developed countries.

Timely and proper treatment can restore part of the visual function of patients with ocular trauma. In our series, 24.7% of patients had final visual acuities <10/200, which may be related to the delayed intervention after occurrence of injuries. Impairment of monocular vision may not lead to loss of labor force, so a lesser degree of eye injuries sometimes will not receive enough attention from patients. In this study, only 45.5% of patients sought medical consultation within 24 hours after the traumatic event. In developed countries, this ratio was reported to be higher than 80% [17, 20]. Another reason for the poor visual outcomes might be the limited experience and expertise of doctors in primary hospitals in China. The choice of surgical procedures and timing is not supported by evidence-based medicine, which can make the injury more complicated. Furthermore, there has been no established consultation and referral system yet. An optimal system should include at least two parts: digital remote consultation based on mobile Internet and timely referral after accurate classification. The optimal therapeutic timing may be missed during the period of referral to different levels of eye care institutions.

Consistent with previous studies, the types of ocular trauma influence the final visual acuity [2, 3, 13]. Compared with closed-globe injuries, open-globe injuries destroy the global structure, causing damage and prolapse of the intraocular contents. In these cases, it is difficult for the patient's visual function to recover. In nonmechanical ocular trauma due to the different injury mechanisms, the damage to the eye tissue is continuous, and patients are often blinded by long-term complications, such as corneal perforation, limbal stem cell deficiency, and secondary glaucoma [21].

It is worth noticing that comparing with other studies, this study contained more nonmechanical injuries, and it can cause data difference with similar studies. For example, the incidence of bilateral eye injuries and the proportion of patients received amniotic membrane transplantation could be higher.

The limitation of this study is that the recorded data of ocular trauma were obtained from a single hospital rather than the regional population. The status of ocular trauma in Shandong Province might not be completely reflected although the hospital is a leading eye center in the region. Meanwhile, there were fewer cases of eye injuries caused by traffic accidents, violence, and other factors combined with facial trauma or systemic injuries in a hospital specialized in eye diseases. However, these limitations do not significantly affect the major findings of this study.

In summary, hospitalized patients with ocular trauma were mainly males at working age in Shandong Province. The injuries usually occurred at the workplace, with a high proportion of nonmechanical trauma. The final visual acuity was influenced by the time that elapsed before visiting hospital and the type of ocular trauma. The final visual acuity of closed-globe injuries is better than open-globe injuries, as well as mechanical injuries and nonmechanical injuries. We should strengthen education on safety protection as well as improve safety facilities and working environment for people who carry a high risk for ocular trauma, thus reducing the incidence of ocular trauma. It is also imperative to reinforce the professional training of eye practitioners in primary hospitals and establish an effective referral system to ensure timely and proper intervention, improve prognoses, and reduce economic burdens for patients with eye injuries.

## Figures and Tables

**Table 1:** Characteristics of patients hospitalized for ocular trauma over a 5-year period at the Shandong Eye Hospital.

Total patients	1,425
Laterality of eyes (%)	
OD	570 (40.0%)
OS	658 (46.2%)
OU	197 (13.8%)
Type of ocular injury	
Mechanical eye injury	944 (66.2%)
Closed-globe injury	454 (31.9%)
Contusion	287 (20.1%)
Lamellar laceration	167 (11.7%)
Open-globe injury	490 (34.4%)
Penetrating	279 (19.6%)
Perforating	2 (0.1%)
Rupture	102 (7.2%)
Intraocular foreign body	107 (7.5%)
Nonmechanical eye injury	426 (29.9%)
Chemical injury	303 (21.3%)
Thermal injury	123 (8.6%)
Accessory injury	55 (3.9%)
Time interval from injury to presentation	
<6 hours	313 (22.0%)
6–12 hours	199 (14.0%)
12–24 hours	137 (9.6%)
1–7 days	354 (24.8%)
≥7 days	422 (29.6%)
Mean duration of follow-up (months)	11.2 ± 2.6

**Table 2:** Nonsurgical and surgical management of eye injuries.

Management	Frequency	Percentage (%)
Nonsurgical	193	12.1
Debridement and suture	540	33.8
Lens extraction	31	1.9
Glaucoma surgery	40	2.5
Posterior vitrectomy	138	8.6
Amniotic membrane transplantation	445	27.9
Canalicular anastomosis	37	2.3
Enucleation	18	1.1
Others	155	9.7
Total	1597	100

**Table 3:** The percentage of the final visual acuity based on the OTS in mechanical injuries.

Raw score	OTS category	NLP (%)	LP/HM	1/200–19/200	20/200–20/50	≥20/40
0–44	1	24	40	19	18	0
45–65	2	2	20	37	32	9
66–80	3	0	3	38	44	16
81–91	4	0	1	4	43	52
92–100	5	0	0	0	6	94

## Data Availability

The data used to support the findings of this study are available from the corresponding author upon request.
